# The Role of Aquaporins in Ocular Lens Homeostasis

**DOI:** 10.3390/ijms18122693

**Published:** 2017-12-12

**Authors:** Kevin L. Schey, Rosica S. Petrova, Romell B. Gletten, Paul J. Donaldson

**Affiliations:** 1 Department of Biochemistry, Vanderbilt University, Nashville, TN 37240, USA; romell.gletten@vanderbilt.edu; 2 Department of Physiology, School of Medical Sciences, New Zealand National Eye Centre, University of Auckland, Auckland 1023, New Zealand; r.petrova@auckland.ac.nz (R.S.P.); p.donaldson@auckland.ac.nz (P.J.D.); 3 School of Optometry and Vison Sciences, New Zealand National Eye Centre, University of Auckland, Auckland 1023, New Zealand

**Keywords:** microcirculation, hydrostatic pressure, regulation, post-translational modification

## Abstract

Aquaporins (AQPs), by playing essential roles in the maintenance of ocular lens homeostasis, contribute to the establishment and maintenance of the overall optical properties of the lens over many decades of life. Three aquaporins, AQP0, AQP1 and AQP5, each with distinctly different functional properties, are abundantly and differentially expressed in the different regions of the ocular lens. Furthermore, the diversity of AQP functionality is increased in the absence of protein turnover by age-related modifications to lens AQPs that are proposed to alter AQP function in the different regions of the lens. These regional differences in AQP functionality are proposed to contribute to the generation and directionality of the lens internal microcirculation; a system of circulating ionic and fluid fluxes that delivers nutrients to and removes wastes from the lens faster than could be achieved by passive diffusion alone. In this review, we present how regional differences in lens AQP isoforms potentially contribute to this microcirculation system by highlighting current areas of investigation and emphasizing areas where future work is required.

## 1. Introduction

Our sense of sight is critically dependent on the optical properties of the lens and age dependent changes to these properties manifest as presbyopia in middle age and cataract in the elderly. The optical properties of the lens are, in turn, a product of its transparency and refractive properties [1] that are both determined by lens tissue architecture and cellular function [2,3,4,5]. The refractive properties are also dependent on overall lens geometry and the presence of a gradient of refractive index (GRIN) [6,7]. The structural properties of the lens are first established early on in embryonic development [8] and are then continually modified as the lens grows throughout life with lens proteins being subjected to age-dependent post-translational modifications [9,10]. To preserve its cellular structure the lens possesses a unique internal microcirculation system that, in the absence of a blood supply, delivers nutrients, removes metabolic wastes and controls the ionic homeostasis of the deeper lens cells [11,12,13]. Since this microcirculation involves the creation of a water flux that circulates throughout the lens, it has been proposed that the differential expression of members of the aquaporin (AQP) family of water channels in these different lens regions plays an important role in the generation of the microcirculation [12]. In this review we investigate how the specific properties of the three AQPs whose expression has been confirmed in the lens, AQP0, AQP1 and AQP5, contribute to the functionality of the different regions of the lens and how these water channels contribute to the generation and regulation of the microcirculation system that actively maintains the overall transparent and refractive properties of the lens [1]. We start our review with an overview of lens structure and function to provide the necessary framework to discuss the specific contributions of each lens AQP in the different lens regions.

## 2. Lens Structure and Function

**Figure 1:**
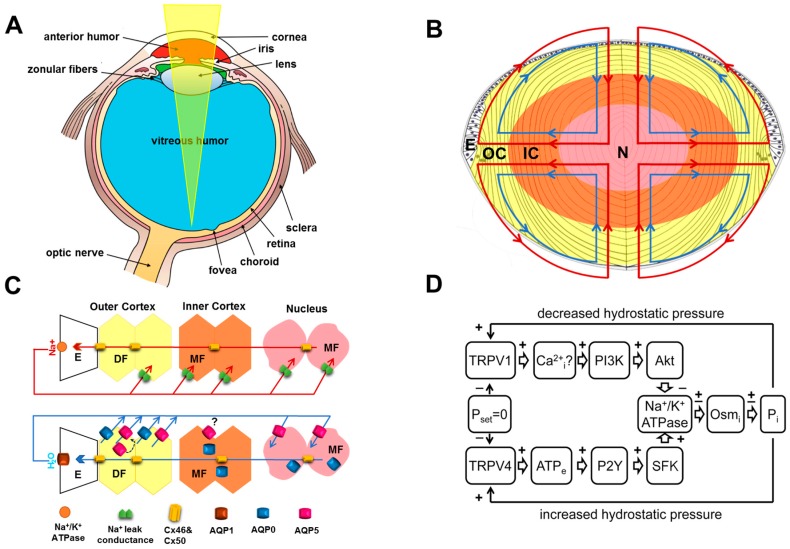
Structure and function of the ocular lens. (**A**) The cornea and the lens work together to focus light onto the retina. In the young primate lens the contraction of the ciliary muscle can alter the tension applied to the lens via the zonular fibers and change the shape and therefore optical power of the lens. This process of accommodation is lost as the lens becomes progressively stiffer through middle age resulting in presbyopia. With advanced age, the lens gradually loses it transparency which can ultimately manifest as cataract. Adapted from Wikimedia Commons contributors. Three Internal Chambers of the Eye.png. Available online https://commons.wikimedia.org/w/index.php?title=File:Three_Internal_chambers_of_the_Eye.png&oldid=223135955 (accessed on 10 November 2017); (**B**) the lens is composed of a single layer of epithelium (E) located at the anterior pole and elongated fiber cells that fill the bulk of the lens. At a narrow region around the equator the epithelial cells undergo differentiation and transform into fiber cells that ultimately become organized into symmetrical growth sheets that stay viable throughout one’s life. The older cells are located in the center or nucleus (N) of the lens while the newly differentiated fiber cells are found in the periphery or outer cortex (OC) of the lens. A net flux of Na^+^ and fluid enters the lens at both poles and exits at the equator to generate an internal microcirculatory system that delivers nutrients to and removes waste products from the avascular lens faster than would be achieved by passive diffusion alone; (**C**) spatial differences in ion transport properties generate the microcirculation. **Top** panel: Na^+^ flows into the lens along the extracellular spaces between cells and crosses fiber cell membranes by diffusing down its electrochemical gradient, then flows back to the lens surface via an intracellular pathway mediated by gap junctions that direct Na^+^ to the equator where the Na^+^/K^+^ ATPase transports it out of the lens to complete the circulation; **Bottom** panel: Water follows the circulating Na^+^ current entering the lens at each pole via the extracellular space before entering fiber cells through AQP0/5 water channels before flowing back to the surface via gap junctions and leaving the lens through AQP1 channels located in the epithelium; (**D**) the movement of water through gap junctions generates a hydrostatic pressure gradient (*Pi*, mmHg) that helps to drive the water from the core to the surface. This hydrostatic pressure gradient is dynamically regulated by a dual feedback system that uses the mechano-senstiveTRPV1 and TRPV4 to sense increases and decreases in pressure, respectively and to activate signaling pathways that reciprocally alter Na^+^, K^+^-ATPase activity to maintain a constant pressure.

The adult human lens is an avascular oblate spheroid that is attached to the ciliary body by a series of fibrillar zonules that hold the lens in a position in the eye between the aqueous and vitreous humors (Figure 1A). At the cellular level the lens is surrounded by a collagenous capsule (Figure 1B). Beneath the capsule a single layer of epithelial cells covers the anterior surface of the lens that interfaces with the aqueous humor of the lens. The bulk of the lens is comprised of fiber cells at different stages of differentiation with the oldest, most differentiated fibers being found in the lens center, or nucleus. The primary fiber cells in the nucleus originate from elongation and differentiation of epithelial cells at the posterior pole of the lens vesicle during early stages of embryonic lens development. The secondary fiber cells originate from anterior epithelial cells at the lens equator and embark upon a process of differentiation that involves extensive elongation and the expression of a variety of fiber cell-specific proteins [2,4]. These differentiating fiber (DF) cells have a distinctive hexagonal profile that facilitates their packing into a crystalline array that minimizes the extracellular space. DF cells continue to elongate until they reach the anterior and posterior poles of the lens where they interface with fibers from the adjacent hemispheres to form the lens sutures [14]. Towards the end of this program of differential protein expression, elongation and migration, DF cells abruptly lose their cellular organelles and nuclei to form mature fiber (MF) cells that occupy the inner cortex and nucleus of the lens [5]. Because this process of fiber cell differentiation occurs throughout life, an inherent age gradient of fiber cells is established with younger secondary DF cells internalizing older MF cells located in the lens nucleus. In fact, the embryonic nucleus contains so called primary fiber (PF) cells that were initially laid down during embryonic development and are retained throughout life [8].

Imposed upon this inherent gradient in fiber cell age is a GRIN that is highest in the MF cells of the lens nucleus and lowest in the peripheral DF cells [15]. This gradient is established by the differential expression of lens crystallin proteins throughout the course of embryonic development and subsequent growth of the lens [16]. It is the GRIN and the surface curvatures of the lens that determine the refractive properties of the lens and set its optical power, i.e. its ability to converge light to a point of focus. However, to focus light, the lens has to be transparent and this is achieved by: the unique cellular architecture of the lens, the absence of blood vessels, the removal of cellular organelles, the minimizing of the extracellular space and a matching of the refractive index between the membranes and the cytoplasm. Together, these features eliminate potential sources of light scattering and therefore establish the transparent properties of the lens [1]. Thus, the optical properties of the lens are direct result of its specialized tissue architecture that is initially established during embryonic development but is continually modified as the lens grows throughout life without the loss of those original PF cells.

The lens, however, is not a purely passive optical element. The adoption of specialized tissue and cellular architecture needs to be actively maintained by a unique physiology in order to maintain transparency for decades without direct access to the blood circulation system [11,12,13]. While the details of how this microcirculation system maintains lens transparency and refraction have not been fully elucidated [17,18]; it is apparent that the AQPs play critical role in the generation of the microcirculation system.

## 3. The Lens Microcirculation System

The metabolic demand imposed on the lens to efficiently transport nutrients and remove waste from deeper lying cellular layers in the absence of an inherent blood supply have been a focus of extensive research. In this regard, Mathias et al. [12,13] have proposed a microcirculation model, in which ion fluxes directed through distinct extracellular and intracellular pathways generate an internal fluid circulation that delivers nutrients to and removes wastes from the lens core (Figure 1C). While not universally accepted by all [17], evidence in favor of the model is accumulating [19,20,21].

It has been proposed that the lens microcirculation is generated by a circulating flux of Na^+^ that enters the lens at both the anterior and posterior poles via the extracellular space between adjacent fiber cells. The Na^+^ eventually crosses the fiber cell membranes and then flows back to the surface of the lens via an intracellular pathway mediated by gap junction channels (Figure 1C). Because gap junctions in the outer cortex of the lens are more abundantly localized at the equator, the intracellular exit pathway is directed towards the lens equator where the highest density of Na^+^/K^+^ pumps (relative to the poles) are located, to complete the circulation of Na^+^ by actively transporting Na^+^ out of the lens [22,23]. The generating power of the electromotive Na^+^ current is established and maintained by the expression of Na^+^/K^+^ pumps localized in the epithelium and newly formed DF cells. Deeper fiber cells lack functional Na^+^/K^+^ pumps and K^+^ channels and a negative membrane potential is maintained in these deeper cells by virtue of their coupling to the surface cells via gap junctions. This electrical connection together with the differential expression of membrane proteins of the superficial and deeper fiber cells causes the standing current to flow. In this model, the circulating current generates an isotonic fluid flow that is directed into the lens via the extracellular space and exits via an intracellular pathway. It is hypothesized that this extracellular flow of water convects nutrients to the deeper lying fiber cells, whereas the intracellular outflow removes waste, transforming the lens into a well stirred compartment. Thus, the electrochemical gradient of Na^+^ ions imposed by the surface cells is not only responsible for the movement of nutrients but also sets the negative membrane potential necessary to maintain the steady state volume of the inner fiber cells [12,24].

The inclusion of water flow into the circulation model dramatically altered the model and led to the prediction that a large intracellular hydrostatic pressure gradient will be generated by the flow of water through gap junction channels (Figure 1C). This hydrostatic pressure gradient has been measured and is thought to drive intracellular flow of fluid from the lens core to the periphery [21]. This hydrostatic pressure gradient, which ranges from 0 mmHg in the periphery to 335 mmHg in the lens center, is remarkably preserved in all species of lenses examined to date [25]. This conservation of the pressure gradient among species has led to the suggestion that the gradient is actively modulated and controls the water content in the lens core to set the water/protein ratio that determines the gradient of refractive index that contributes to the optical properties of the lens [26].

Maintaining constant water flow/hydrostatic pressure requires that the activities of all transport proteins involved in generating the ionic and fluid fluxes that drive the microcirculation are synchronized. In this regard the regulation of the Na^+^/K^+^ ATPase in the lens has been well studied [27]. Shahidullah et al. has shown that in response to hyposmotic challenge TRPV4 channels are activated by the change in cell volume and initiate a signaling cascade that involves the release of ATP into the extracellular space via connexin hemichannels (Figure 1D). The activation of ATP sensitive P2Y receptors increases the activity of a Src family kinase (SFK) that ultimately increases Na^+^/K^+^ ATPase activity [27]. Conversely, Sellitto et al. have shown that Na^+^/K^+^ ATPase activity in the lens can be decreased through a mechanism dependent on PI3K (Phosphoinositide 3-kinase)/Akt (Protein kinase B) signal transduction pathway in lens epithelial cells [28], although the upstream activation of this pathway was not identified in this study. Interestingly, in other tissues TRPV1 has been shown to play an opposing role to TRPV4 in osmoregulation [29,30] and TRPV1 is known to be expressed in lens epithelial cells [31]. Subsequently, it was shown that the application of specific TRPV1 and TRPV4 agonists to the mouse lens resulted in initial decreases and increases in hydrostatic pressure, respectively [26]. These results suggest that TRPV1 and TRPV4 act as the initial sensors of two opposing arms of a feedback system that utilize distinct signaling pathways to keep lens hydrostatic pressure constant by modulating Na^+^/K^+^ ATPase activity (Figure 1D).

In this scheme, it is the active transport of Na^+^ that drives a directed isotonic flow of fluid through the lens. Isotonic fluid flow, in turn, requires a high membrane permeability to water that is known to be mediated by the aquaporin (AQP) family of water channels. However, what is not known is how do the different lens AQPs, which exhibit distinctly different functional properties and expression patterns, contribute to the generation of the microcirculation system. To address this, we first compare the expression patterns of three lens AQPs before detailing their functional properties in order to develop a working model on how the different AQP isoforms contribute to the generation of the lens microcirculation system that is so central to the maintenance of lens homeostasis.

## 4. Regional Expression Patterns of Lens Aquaporins during Development and Growth

The expression of AQP0, AQP1 and AQP5 is conserved in all mammalian lenses examined thus far [32,33]. A fourth member of the aquaporin family, the aquaglyceroporin AQP7, has also been detected in the human lens via immunohistochemistry [34]. AQP7 regulates energy metabolism in several cell types suggesting the potential for AQP7 to perform a similar role in the ocular lens [35,36]. However, given the lack of additional reports of lenticular AQP7 expression, the role of AQP7 will not be discussed in the remainder of this review. Since AQP0, AQP1 and AQP5 exhibit distinct water permeabilities and regulatory mechanisms [37,38], the conservation of the regional expression patterns of these three AQPs isoforms across all mammalian lenses studied to date implies unique contributions to overall lens homeostasis. These differences in expression are initiated during development and are maintained into adulthood.

**Figure 2:**
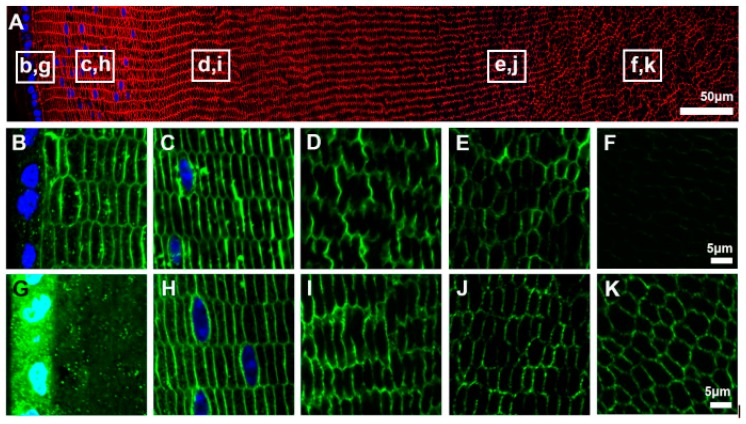
Comparison of the subcellular expression patterns of AQP0 and AQP5 in the rat lenses. (**A**) An equatorial section of a 6-week-old rat lens labeled with the membrane marker WGA (red) and the nuclear stain DAPI (blue) showing the regions (white boxes) from which high magnification images of the subcellular location of AQP0 (**b**–**f**) and AQP5 (**g**–**k**) were obtained; (**B**–**F**) AQP0 (green) is expressed in the membrane of both differentiating fiber cells and mature fiber cells but the antibody labelling is lost in the lens core (**F**) due to truncation of the C-terminal tail of AQP0 which contains the antibody epitope; (**G**–**K**) AQP5 labelling is associated with the membranes of fiber cells throughout the majority of the lens except for a region in the lens periphery where it is found as a pool of cytoplasmic labelling (**G**) before it translocates exclusively to the membranes of differentiating fiber cells (**H**).

During development, AQP0 expression is first detected at E11, while AQP5 is present at E10 but only in the cell cytoplasm [39]. This subcellular distribution of AQP5 suggests that it does not contribute to plasma membrane water permeability at this early developmental stage, when the lens is nourished by the hyaloid vascular system (HVS). This result is in agreement with a previous study suggesting that the lens microcirculation system is not initiated until later stages of embryonic development when AQP1 protein is found to initiate its expression in the lens embryonic epithelium at embryonic day E17.5 [40]. The authors of the same study proposed that AQP0 serves as a mainly structural protein during lens embryonic development to facilitate formation of the ordered cellular architecture of the adult lens, while during postnatal development it serves mainly as a water channel to maintain homeostasis of the growing lens. From P6 to P15 changes in lens AQP distribution occurs when AQP0 undergoes a developmentally programmed C-terminal truncation in the core of the lens which coincides with the insertion of AQP5 into the plasma membrane [39]. These changes in the localizations of the AQPs occur during the period when the HVS begins to regress [41]. The loss of the HVS results in a switch from the lens being a vascular tissue to being an avascular tissue that is solely dependent on the fluid microcirculation system to maintain transparency after eye opening. After eye opening the patterns of AQP expression established during the embryonic and post-natal periods of development and growth are maintained into adulthood. While AQP1 is confined to the epithelium, the relative expression patterns of AQP0 and AQP5 in the different regions of the adult lens are distinctly different (Figure 2) and suggest different roles for the two AQPs in the different regions of the lens. These potential roles are discussed for each AQP below.

*(i) AQP1 expression.* AQP1 is a ubiquitous, constitutively open water channel [42] that in the human and rodent lens is expressed in the apical and basolateral membranes of the epithelial cells [33,40,43]. AQP1 expression progressively increases during postnatal development and growth which coincides with an increase in the size of the lens [40]. Deletion of AQP1 in the lens epithelium resulted in an approximately threefold reduction of the water permeability of AQP1 knockout mice lenses [44]. The same study reported development of accelerated lens opacities in both in vitro organ cultured AQP1 knockout lenses incubated in high glucose solution and in in vivo conditions after acetaminophen treatment used to induce formation of lens cataract in comparison to the counterpart wild type non-treated lenses. Furthermore, it has recently been shown that patients with cataract showed an increased level of membrane expression of AQP1 in lens epithelial cells although no specific molecular mechanisms were described that were involved in this elevated levels of membrane expression [45]. Thus, AQP1 is required to maintain the transparency of the lens, especially following exposure to stress conditions such as hyperglycemia and osmotic imbalance.

*(ii) AQP0 expression.* As lens fiber cells differentiate from epithelial cells at the lens equator AQP1 expression is shut down and AQP0 expression is turned on in the newly formed secondary fiber cells. AQP0 then becomes the most abundant integral membrane protein in lens fiber cells comprising approximately 50% of total lens membrane protein [46]. Initially thought to be uniquely expressed in lens fiber cells, AQP0 has now been reported in retina [47], liver [48] and testes [49]. Although highly abundant, AQP0 was determined to be a weak water channel with a water permeability 30-fold lower than AQP1 and approximately 20-fold lower than AQP5 [37,50,51,52]. Unlike AQP1 and AQP5, AQP0 is not sensitive to mercury compounds [51] due to the absence of a mercury sensitive cysteine residue in the water pore.

AQP0 is unique among the aquaporins for its multifunctional role in lens fiber cells. In addition to being a water channel, AQP0 has adhesion properties that are lacking in the other lens AQPs [53]. This cell-to-cell adhesion (CTCA) property is presumably due to interactions of AQP0 with either the lipid head groups of adjacent cells, or with APQ0 molecules of opposing membranes. Indeed, a structural model of these interactions was first proposed by Lo and Harding [54] showing that in mature fiber cells, AQP0 forms wavy thin (10 nm) junctions or square array junctions between adjacent fiber cells which was later adopted by others [55,56,57]. Note that replacement of AQP0 in knockout mice with AQP1 does not fully rescue the cataract phenotype suggesting essential CTCA properties present in AQP0, are lacking in AQP1. This dual role of AQP0 is further exemplified in zebrafish in which an evolutionary duplication of the AQP0 gene resulted in sub-functionalization into two isoforms: AQP0a functions as a water channel, while AQP0b is not a water channel but has a potential adhesive role, although this has yet to be confirmed. Deletion of either isoform, results in the formation of cataract, demonstrating that both isoforms and therefore both functions, are required for the normal development and transparency of the lens [58,59,60]. An additional function of AQP0 in fiber cell membrane organization and cell structure is suggested by interactions with lens specific cytoskeletal proteins, filensin and phakinin [61,62] and cytoskeletal linker proteins [63]. In AQP0-null mice gap junction area increased and fiber cell shape and lateral interdigitations/protrusions were significantly altered [64,65].

There are several mutations to AQP0 that result in congenital cataract. In mice, a mutation known both as *Cat^Fr^* (named for the cataract Fraser mouse in which it appears) and AQP0-LTR (named as a description for the mutation to AQP0) results in the replacement of the C-terminus of AQP0 with a long terminal repeat (LTR), such that amino acids 203–261 are different in AQP0-LTR [66]. AQP0-LTR fails to traffic to the membrane and, by 3 weeks of age, there is bilateral cataract with an anterior sub-polar opacity in the homozygous AQP0-LTR lenses [67]. Other AQP0 mutations in mice include the *Lop* [66], *Cat^Tohm^* [68] and the *Hfi* [69] strains. Each of these mutant AQP0 proteins fails to traffic to the plasma membrane resulting in bilateral cataract. In humans, seven mutations to AQP0 have been detected that result in cataracts and all result in failed trafficking of AQP0 to the fiber cell plasma membrane. These mutations include Glu134Gly [70], Thr138Arg [70], Arg233Lys [71], Arg33Cys [72], Asp150His [73] as well as C-terminal truncation mutants, Δ213-AQP0 [74,75] and Tyr219stop [76]. 

AQP0 clearly plays a role in establishing and maintaining lens optical properties as evidenced by cataract formation in mutant AQP0 lenses described above and by distorted focal properties in lenses from AQP0 knockout animals [77]. In addition, lens biomechanical properties are also altered in lenses from AQP0 but not AQP5, knockout animals suggesting a role for AQP0-cytoskeleton interactions in presbyopia [78]. Gerometta and Candia also argue that a decrease in the permeability of AQP0 could be a cause of presbyopia [79].

*(iii) AQP5 expression.* Indications that the lens may express a third water channel surfaced when AQP5 mRNA was detected in rat [33] and human [80] lenses. Later proteomic approaches identified AQP5 at the protein level in fiber cells from both the mouse and bovine lens [81,82]. The expression of AQP5 in the adult lens was subsequently confirmed by multiple laboratories [32,83]. AQP5 is expressed in both epithelial cells and lens fiber cells. AQP5 expression in lens fiber cells is significantly lower (<5%) than AQP0 [81,82]. However, AQP5 has significantly greater water permeability compared to AQP0 (~20 fold) [37]. Immunohistochemical studies in human and rodent lenses demonstrate distinct AQP5 subcellular localization in comparison to AQP0 (Figure 2) [32,39]. AQP5 is cytoplasmically expressed in the epithelial and younger differentiating fiber cells of the lens outer cortex. This cytoplasmically localized AQP5 has been recently found to differentially translocate to the membranes of lens epithelial cells in patients with cataract which the authors suggest may serve as a compensatory mechanism to reduce the severity of cataract [45]. As fiber cells progressively age from the outer cortex to the nucleus, AQP5 abruptly translocates to the plasma membrane. In the older mature fiber cells of the inner lens nucleus, AQP5 immunostaining on the plasma membrane predominates and becomes undetectable in the cytoplasm.

Taken together the different labelling pattern observed for AQP5 compared to AQP0 [84] raises questions about the relative functional roles of the two AQPs in the different regions of the lens. Initial characterization of lenses from AQP5 knockout animals showed that in comparison to wild type lenses, organ cultured knockout lenses developed cataract after 60 h incubation in high glucose (55.6 mM) media. [85]. Moreover, the spatial correlation between AQP5 plasma membrane insertion and AQP0 C-terminal truncation in whole lens tissue sections is notable. AQP0 C-terminal truncation induces formation of thin junctions, which are predominately localized to the lens nucleus [55,56,86]. Electron crystallographic data suggests that AQP0 permeability is reduced by thin junction formation [86]. Together, these data implicate a compensatory role for AQP5 in mitigating reduced AQP0 water permeability in the lens core. A clearer understanding of the cellular mechanisms that initiate and regulate AQP5 plasma membrane insertion and gating in the lens is vital to more thoroughly define lenticular AQP5 function.

## 5. Regulation of AQP0 and AQP5 Function in Fiber Cells

Since AQP0 and AQP5 are expressed in both DF and MF cells it would be expected that their functional properties and the regulation of that function, would change as the signaling pathways, association with interacting proteins, lipid environment and post-translation modifications, that all alter the function of AQP0 and AQP5 would change in the different regions of the lens. Before examining how these changes potentially contribute to the generation of the microcirculation system the regulation of AQP0 and AQP5 is first reviewed below.

*(i) Regulation of AQP0.* AQP0 water permeability has been shown to be regulated by calcium, pH, and phosphorylation. Specifically, the water permeability of AQP0 can be increased 2-fold by incubating vesicles in high Ca^2+^ or low pH [68]. The sensitivity to external pH is attributed to a histidine residue (His 40) localized in the first extracellular loop, whereas sensitivity to Ca^2+^ is determined by the interaction of the C-terminal domain of AQP0 with calmodulin. The combination of high Ca^2+^ and low pH did not have a cumulative effect on water permeability, suggesting that the two regulatory sites worked through a common mechanism, either by increasing the open probability of the water channel or by increasing the open-channel permeability [68]. These findings were generally consistent with results obtained by exogenously expressing bovine AQP0 in oocytes to study the regulation of AQP0 water permeability [87]. However, the elevation of Ca^2+^ had opposite effects in the two experimental systems. In oocytes, the elevation of extracellular Ca^2+^ resulted in a decrease of membrane water permeability. This difference is thought to be due to both the different membrane composition of oocytes and lens fiber cell vesicles and differences in the affinity of apo-calmodulin and Ca^2+^-calmodulin to the C-terminus domain of AQP0 in the native (lens) and exogenous (oocytes) systems. Recently, an additional regulatory site of the AQP0-calmodulin interaction was reported in the arginine-rich intracellular loop [88].

Several sites of AQP0 phosphorylation have been identified [89]. Phosphorylation of AQP0 at Ser235, the most abundant phosphorylation site, occurs via PKA and is mediated by AKAP2 [90]. The phosphorylation of the C-terminal tail has been shown to disrupt calmodulin binding [89,91] and increase AQP0 permeability in the presence of elevated Ca^2+^. Interestingly, quantitative analysis of phosphorylated AQP0 shows that it reaches a maximum in the inner cortex of the young human lenses, before decreasing in older lenses [92]. It is interesting to speculate that this decrease AQP0 phosphorylation in the inner cortex of older lenses reduces fiber cell water permeability in a region where a barrier to water transport has been demonstrated [93,94].

Protein lipidation is known to regulate protein function by modulating membrane targeting, protein-protein interactions and lipid raft domain targeting. AQP0 is modified by palmitoylation (C16:0, +238 Da) and oleic acid (C18:1, +264 Da) at N-terminal M1 and C-terminal K238 in bovine and human lenses [95]. Other fatty acid modifications to AQP0 have also been reported [96]. Non-lipidated AQP0 was detected in detergent-soluble membrane fractions (DSM), whereas lipidated AQP0 was detected exclusively in detergent-resistant membrane (lipid raft) fractions. Furthermore, the spatial distribution of lipidated AQP0 is distinct, occurring in highest abundance in the inner cortex region [95].

Given the lack of protein turnover in the lens, lenticular AQPs undergo extensive age-related modifications [97]. These modifications include extensive truncation, deamidation, and racemization. Truncated AQP0 has been shown to form AQP0-AQP0 junctions with predicted low water permeability due to a closure of the water pores [57,86]. In contrast, when truncated AQP0 proteins were expressed in oocytes [98,99], no difference in water permeability was measured compared to full length AQP0. Since the AQP0 C-terminus is a site of protein-protein interactions with calmodulin [89,91,100] and cytoskeletal proteins [61,62], age-related C-terminal truncation is expected to impair such interactions. The functional consequences of this loss remain to be examined. Furthermore, the functional consequences of C-terminal deamidation and racemization also remain to be determined.

*(ii) Regulation of AQP5.* Immunolabeling of lens sections has shown that AQP5 in peripheral fiber cells is predominantly cytoplasmic before transitioning to the plasma membrane in deeper lying fiber cells. In addition to this differentiation-dependent insertion of AQP5 into the membrane, Petrova et al. have recently observed a dynamic insertion of AQP5 into the membrane of peripheral fiber cells in lenses that have been first subjected to organ culture [101]. In this study, they also showed that this insertion of AQP5 was correlated with a significant increase in a Hg^2+^-sensitive contribution to P_H2O_. Based on these results Petrova et al. suggested that the dynamic shuttling of AQP5 water channels to and from the plasma membrane, can alter the P_H2O_ of peripheral fiber cells in the outer cortex and, therefore, up or down-regulate the water fluxes that exit the lens at the equator.

While the regulatory mechanism(s) that control the shuttling of AQP5 to and from the lens fiber cell membrane has yet to be identified, there is accumulating evidence in other tissues that a variety of signaling pathways can modulate water permeability by increasing the expression of AQP5 and its insertion into the plasma membrane. Similar to the shuttling mechanism described for AQP2 in the renal collecting duct [102], AQP5 has been found to insert into the apical plasma membranes of murine lung epithelial cells (MLE-12) following phosphorylation through the cAMP-dependent PKA pathway [103]. Sequence analysis has suggested that AQP5 possesses two PKA consensus sites within Loop D (aa 153–157) and its C-terminal domain (aa 256–259), with AQP5 S156 and AQP5 T259 representing the predicted phosphorylation sites in the two regions, respectively. In support of this prediction phosphorylated T259 has been recently detected in the lens [104], which suggests by analogy that modulation of the phosphorylation status of AQP5 may be involved in regulating its trafficking to the membrane of lens fiber cells.

In addition to the cAMP-dependent PKA pathway, a number of other stimuli have been shown to increase water permeability by increasing AQP5 expression at the mRNA and protein levels and via increased membrane insertion. Exposure to hypertonic challenge was shown to up-regulate AQP5 protein expression through an Extracellular signal-Regulated Kinase (ERK) dependent pathway in mouse lung epithelial (MLE-15) cells [105]. Additionally, a cholinergic stimulation of mouse parotid (salivary) glands, has been found to increase salivary secretion and translocation of AQP5 to the apical membrane.

AQP5 function may also be regulated by protein-protein interactions (PPIs) which in turn may be modulated by C-terminal phosphorylation; an established mechanism by which AQP-PPIs are modulated [89,106,107]. As described above, AQP0-calmodulin interactions are affected by phosphorylation [89,91]. In addition, PKA directly phosphorylates AQP2 at S256 inducing the translocation of cytoplasmic AQP2-containing vesicles to the apical plasma membrane in the kidney [108]. In MDCK cells transfected with wild-type AQP2, the interaction between AQP2 and multiple interacting proteins associated with trafficking (e.g. annexin-2, clathrin, dynamin, G-actin, Hsc70, and protein phosphatase 1C (PP1C)) is significantly reduced with forskolin-mediated PKA activation [107]. Interestingly, AQP5 T259 is homologous to AQP2 S256 and the C-terminus of AQP5 is also a binding site for AQP5 interacting proteins. A synthetic AQP5 C-terminal peptide (mouse AQP5 251–265) specifically interacts with prolactin-inducible protein and murine urinary protein 4 (Mup4) in ICR and NOD mouse lacrimal gland homogenates, respectively [109]. Antisense oligonucleotide mediated knockdown of prolactin-inducible protein in ICR mice appears to reduce AQP5 plasma membrane localization, although these results require validation [109]. Together, these studies suggest that AQP5 plasma membrane localization may be dynamically regulated by AQP5 C-terminal domain protein-protein interactions. Furthermore, these studies also suggest that reversible AQP5 T259 phosphorylation may modulate AQP5-protein interactions. Protein lipidation is known to regulate protein function by modulating membrane targeting, protein-protein interactions and lipid raft domain targeting. Again, in other tissues such as the salivary gland cells, AQP5 has been known to shuttle between lipid raft and non-raft domains [110,111]. Palmitoylation is a form of reversible lipidation previously demonstrated to regulate protein targeting to and from lipid rafts [112] and AQP5 palmitoylation of AQP5 in the lens has recently been observed (Schey, unpublished data). In addition, quantitative analysis of the lens lipid raft proteome showed that AQP5 is enriched in lipid rafts [113].

## 6. Functionally Distinct Roles of AQPs in the Microcirculation System: A Working Model

**Figure 3:**
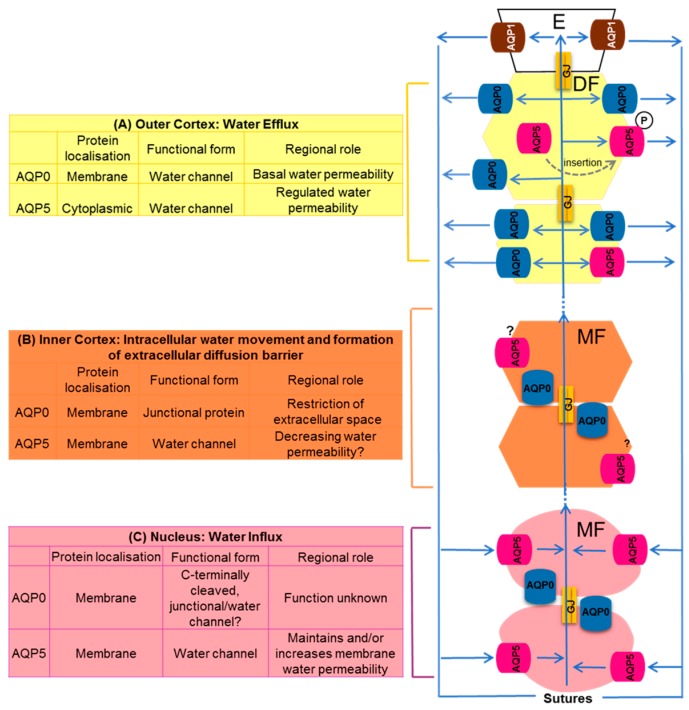
Schematic diagram detailing the relative contributions of AQP0 and AQP5 to fluid circulation in the lens. (**A**) On the anterior surface of the lens cell AQP1 contributes to the water permeability (P_H2O_) of the epithelial cells. At the equator in the outer cortex of the lens basal water efflux is proposed to be mediated by water channels formed from the abundant AQP0. However, in this region of the lens water efflux can be up regulated by the shuttling of AQP5 water channels from an inactive cytoplasmic pool of channels to the membrane to form active water channels following phosphorylation by activation of yet to be identified signaling pathways in the lens; (**B**) in the inner cortex water fluxes are preferentially carried away from the lens core via an intracellular pathway mediated by gap junction channels. In this region AQP0 functionality shifts from being a water channel to a junctional protein that reduces P_H2O_ and narrows the extracellular space to form a barrier to extracellular diffusion. In this region, it is also proposed that the functionality of AQP5 may also be altered to reduce the P_H2O_ of fiber cell non-junctional membranes to facilitate the cell-to-cell movement of water via the gap junctions; (**C**) in the core of the lens mature fiber cells accumulate fluid delivered to them via the sutures. In this region, the C-terminus of AQP0 is cleaved, while AQP5 remains uncleaved and membranous. In this region, the post-translational changes to AQP0 are expected to alter the regulation of AQP0 and therefore AQP5 may compensate for any loss in the contribution of AQP0 to the P_H2O_ of mature fiber cells.

Since water fluxes predicted by the lens microcirculation model [12,13,18] have recently been experimentally confirmed [19,20,21] and been shown to be dynamically regulated [26] it is now important to consider how the observed changes in the subcellular localization and functionality of AQPs in different regions of the lens contribute to the magnitude, directionality and regulation of the water fluxes generated by the microcirculation system. To facilitate this, we have formulated the working model shown in Figure 3. In this model, AQP1 produces a constitutively active water permeability to the epithelial cells, while changes in the subcellular distribution, regulation and post-translational modification of AQP0 and AQP5 in fiber cells in the different regions of the lens alter the relative contributions of the two AQPs to the circulating water flux.

In this model, the water efflux in the outer cortex of the lens is mediated by the constitutively expressed AQP0 in fiber cell membranes that provides a basal water permeability in peripheral fiber cells (Figure 3A). This basal water permeability can be either increased or decreased by the dynamic insertion or removal of AQP5 from the membranes of peripheral DF cells, respectively, possibly by a PKA dependent pathway [83]. In the inner cortex (Figure 3B), the redistribution of AQP0 protein in the plasma membrane results in the formation of junctional structures that are suggested to be involved in restriction of the extracellular space to create an extracellular diffusion barrier [84]. In this region of the lens it is proposed that water transport is predominately directed from cell to cell via gap junction channels and it is proposed that the membrane water permeability of the MF cells in this region is reduced through closure of AQP water channels to facilitate the outflow of water in this region via this intracellular pathway. In the core of the lens AQP0 undergoes extensive cleavage to its C-terminus that we expect would change its water permeability and/or junctional properties (Figure 3C). Since AQP5 does not exhibit the same extent of post translational modification and is 20X more permeable to water than AQP0 [37], it is interesting to speculate that in this central region of lens AQP5 maintains or even increases water permeability allowing water to enter the MF cells in the core from the extracellular space. Since it is a working model our interpretation of the relative roles of AQP0 and AQP5 in the different regions of the lens raises a number of predictions and unanswered questions.

## 7. Predictions and Unanswered Questions

Since the flow of water through gap junction channels generates a gradient in hydrostatic pressure, which we now know is regulated by a complex dual feedback system (Figure 1D), that is designed to maintain a constant pressure gradient so that water is actively removed from the lens core [26], thereby preserving the GRIN and thereby maintaining the optical properties of the lens [114]. While current studies into the regulation of this pressure gradient to date have focused on the modulation of the Na^+^/K^+^ ATPase activity which generates the circulating ion fluxes that drive the microcirculation, we would predict from our model that parallel changes to the water permeability of DF cells at the lens surface would also be required to maintain a constant internal hydrostatic pressure in response to changes in Na^+^ pump rate. Interestingly, an emerging synergy between TRPV4 channels and AQP water channels is emerging in other cell types [115,116,117]. Thus, it is interesting to speculate that TRPV1/4 channel activation in response to changes in hydrostatic pressure may provide a link between changes in Na^+^/K^+^ ATPase activity, the modulation of water permeability in peripheral fiber cells, and the dynamic maintenance of the optical properties of the lens. Thus it would appear that the observed dynamic insertion of AQP5 into the membrane of DF cells in response to lens organ culture [101] may be in response to changes in the hydrostatic pressure gradient that are sensed by TRP channels that, in turn, activate signaling pathways that alter both ion transport and water permeability in parallel to ensure the optical properties of the lens remain constant.

Hall and Mathias [118] describe the AQP0 puzzle wherein they show that the high abundance of lenticular AQP0 is more than sufficient for accommodating fluid circulation in the lens such that a 50% reduction in permeability in AQP0^+/−^ lenses has no effect on hydrostatic pressure. The puzzle, therefore, is what is the role for water permeability and what are the other functions for AQP0? What is the role for AQP5 under these conditions?

Our current model while suggesting specific functions for the AQPs in the different regions of the lens does not capture how these functions change with advancing age. Hence one major unanswered question to emerge from our model is how do the observed age-related modifications to lens AQPs impact on their function? It is possible that the age-dependent modification of AQPs could contribute to the development of the barrier to intracellular diffusion in the inner cortical region of the lens observed in middle age, the formation of which has been implicated in the presbyopia and cataract formation. Obviously, these are all important questions for future study and our working model can serve as a tool to highlight not only that regional differences in AQP functionality exist but also how changes in AQP function can influence the overall structure and function of the lens.

## 8. Conclusions

In conclusion, lens AQPs play critical roles in lens development and homeostasis. With diverse functional properties such as water permeability, cell adhesion and structurally as a membrane anchor for cytoskeletal proteins, loss of AQPs due to mutation or age-related modification results in lens dysfunction. Furthermore, the existence of a dynamic, regulated microcirculation system that can alter lens optical performance is both exciting and far reaching, providing opportunities to study the regulation of lens water transport in more detail at the cellular and molecular levels. The roles of all lenticular AQPs in the lens microcirculation system require further investigation to more clearly elucidate their relative contributions in the different regions of the lens to overall function in both the normal and cataractous lens.
